# NEK7 Coordinates Rapid Neuroinflammation After Subarachnoid Hemorrhage in Mice

**DOI:** 10.3389/fneur.2020.00551

**Published:** 2020-07-08

**Authors:** Gen Li, Yushu Dong, Dongdong Liu, Zheng Zou, Guangzhi Hao, Xu Gao, Pengyu Pan, Guobiao Liang

**Affiliations:** ^1^Department of Neurosurgery, General Hospital of Northern Theater Command (General Hospital of Shenyang Military Command), Shenyang, China; ^2^Dalian Medical University, Dalian, China

**Keywords:** subarachnoid hemorrhage, microglia, NEK7, NLRP3, neuroinflammation, blood-brain barrier

## Abstract

**Background:** Subarachnoid hemorrhage (SAH) is a devastating disease which leads to high morbidity and mortality. Recent studies have indicated that, never in mitosis gene A-related expressed kinase 7 (NEK7), is involved in NLRP3 (NLR family, pyrin domain containing 3) associated inflammation, which may result in subsequent cellular and vascular damage. The aim of this study was to investigate whether NEK7 is involved in the pathophysiology of subarachnoid hemorrhage.

**Methods:** 455 adult male C57B6J mice, weighing 22 to 30 g, were used to investigate the time course of NEK7 expression in the ipsilateral cortex after SAH, and to investigate the intrinsic function and mechanism of NEK7. A vascular puncture model was used to create the mouse SAH model, and intracerebroventricular injection was used to deliver NEK7 recombinant protein, NEK7 small interfering RNA, nigericin, and MCC950. Neurological score, brain water content, Evans blue extravasation, immunofluorescence, and western blot were evaluated for neurological outcome, neuronal apoptosis, blood-brain barrier damage, microglia accumulation, and the mechanism of NEK7 and NLRP3 activation.

**Results:** Our results exhibited that intrinsic NEK7 was elevated after SAH in the cortex of the left/ipsilateral hemisphere and was colocalized with microglia, endothelial cells, neuron, astrocyte, and oligodendrocyte, and highly expressed in microglia and endothelial cells after SAH. NEK7 recombinant protein aggravated neurological deficits, brain edema, neuronal apoptosis, BBB permeability, microglial accumulation, and activated caspase-1 and IL-1β maturation, while NEK7 small interfering RNA injection reversed those effects. Nigericin administration enhanced ASC oligomerization, caspase-1 and IL-1β maturation without increasing the protein level of NLRP3, and ASC oligomerization and caspase-1 IL-1β maturation reduced when combined with NEK7 knockdown or MCC950 delivery. We found the level of NEK7 expression increased after SAH and could activate the downstream NLRP3 pathway to induce caspase-1, IL-1β expression and then increased the BBB opening, microglia accumulation and neuronal apoptosis after SAH.

**Conclusions:** This study demonstrated for the first time that NEK7 mediated the harmful effects of neuronal apoptosis and BBB disruption after SAH, which may potentially be mediated by the NEK7/NLRP3 signal. NEK7 served as a co-component for NLRP3 inflammasome activation after SAH. NEK7 may be a promising target on the management of SAH patients.

## Introduction

Despite years of efforts, early brain injury—the main contributor to the mortality and poor prognosis of patients after subarachnoid hemorrhage (SAH)—remains a catastrophic complication of a ruptured intracranial aneurysm (1). The pathophysiology of SAH involves an alteration in the brain neural vascular network, involving the arterial, capillary, venous systems, neurons, microglia, surrounding support cells, and the extracellular matrix (2). The precise mechanism of neuronal apoptosis and BBB damage, however, remains elusive.

The NLR family, pyrin domain containing 3 (NLRP3) inflammasome, consists of NLRP3, apoptosis-associated speck-like domain (ASC) and pro-caspase-1. It is a group of innate immune proteins considered to be sensors of pathogen- and damage- associated molecular patterns (3). NLRP3 inflammasome activation promotes the cleavage of pro-caspase 1 and pro- interleukin (IL)-1β into their mature forms, which contributes to the development of type 2 diabetes (4), atherosclerosis (5), gout (6), and Alzheimer's disease (7). Our previous study showed NLRP3 activation increased BBB extravasation, tight junction protein degradation, as well as neuronal apoptosis after CNS injury (8, 9). As a typical inflammation target, the exact mechanism of NLRP3 activation still remains unclear.

Recent studies reported that NEK7, a serine and threonine kinase involved in mitosis, is an essential activator of the NLRP3 inflammasome (10). NEK7-mutant cells showed lower caspase-1 activation and IL-1β production in response to NLRP3-activating stimuli (11). These data suggested that NEK7 acts as a switch between inflammasome activation and cell division (11). We postulated that NEK7 expression was altered after SAH and targeting NEK7 might inactivate NLRP3-inflammasome and downstream IL-1β to preserve neuronal death and BBB permeability. To elucidate the function of NEK7 after SAH, we analyzed the spatial distribution and time course of NEK expression, neurological function, neuronal apoptosis, and BBB permeability after NEK7 interference, and downstream NLRP3 activation.

## Materials and Methods

### Animals

All experimental procedures were approved by the Ethics Committee of the General Hospital of Norther Theater Command (Shenyang Military Command) and was performed in accordance with the guidelines in the National Institutes of Health Guide for the Care and Use of Laboratory Animals, and followed the ARRIVE guidelines. Four-hundred-and-fifty-five (455) adult male C57B6J mice (22 to 30 g), were provided by the Department of Experimental Animals of the General Hospital of Norther Theater Command (Shenyang Military Command, Shenyang, China). Mice were acclimated in a reversed 12 h dark/12 h light cycle environment and provided with free access to water and food.

### Experimental Design

The present study contained three experiments, which were designed as follows.

### Experiment I

To investigate the time course of NEK7 expression and cell type location of NEK7 after SAH, 44 mice were randomly assigned to seven groups: Sham (*n* = 6), SAH 3 h (*n* = 6), SAH 6 h (*n* = 6), SAH 12 h (*n* = 6), SAH 24 h (*n* = 6), SAH 48 h (*n* = 6) and SAH 72 h (*n* = 6). NEK7 protein expression was detected by Western blot in cortex isolated from the ipsilateral/left hemisphere. Immunohistochemical staining of NEK7, NeuN (neuron marker), GFAP (astrocyte marker), Lectin (endotheliocyte marker), Iba-1 (microglia marker), and NG2 (oligodendrocyte marker) was performed at 24 h post SAH induction to confirm the spatial distribution of NEK7 in the cortex (*n* = 6).

### Experiment II

To define the intrinsic function of NEK7 and to screen for effective dosage of NEK7 recombinant protein after SAH, 162 mice were randomly assigned to eight groups: Sham (*n* = 24), SAH (*n* = 18), SAH+NS (normal saline, 2 μL) (*n* = 24), SAH+NEK7 5 ng/μL (*n* = 12), SAH+NEK7 25 ng/μL (*n* = 12), SAH+NEK7 100 ng/μL (*n* = 24), SAH+Scr (scrambled) siRNA (*n* = 24), SAH+NEK7 siRNA (*n* = 24). Beam balance and modified Garcia tests were performed at 24 and 72 h after SAH to assess the neurological deficits in each group (*n* = 6). Furthermore, brain water content was performed at 24 and 72 h post SAH. Evans blue extravasation assessment test at 24 h post SAH induction to evaluate the blood-brain barrier damage. Immunohistochemical staining was also performed to detect the neuronal apoptosis, endothelial continuity, and microglia accumulation in the cortex of ipsilateral/left hemisphere at 24 h after SAH induction (*n* = 6).

Furthermore, 30 mice were randomly divided into the following groups: Sham (*n* = 6), SAH+NS (*n* = 6), SAH+NEK7 100 ng/μL (*n* = 6), SAH+Scr siRNA (*n* = 6), SAH+NEK7 siRNA (*n* = 6). Western blot was performed to detect the NEK7, NRLP3, ASC, caspase-1, IL-1β, BCL-2, and BAX expression in cortex of the ipsilateral/left hemisphere in each group.

### Experiment III

To validate the function of NEK7-denpdent NLRP3 signal activation after SAH induction, 84 mice were assigned to seven groups: Sham (*n* = 12), SAH (*n* = 12), SAH+Vehicle (*n* = 12), SAH+nigericin (*n* = 12), SAH+nigericin+Scr siRNA (*n* = 12), SAH+nigericin+NEK7 siRNA (*n* = 12), and SAH+MCC950 (*n* = 12). Modified Garcia tests and beam balance were performed at 24 h post SAH to evaluate the neurological dysfunction and brain water content, and Evans blue extravasation assessments were carried out to test the blood-brain barrier permeability.

Furhermore, 72 mice were randomly divided into the following groups: Sham (*n* = 6), SAH+NS (*n* = 12), SAH+Ngericin (*n* = 12), SAH+ Ngericin+Scr siRNA (*n* = 12), SAH+Ngericin+NEK7 siRNA (*n* = 12), and SAH+MCC950 (*n* = 12). Immunohistochemical staining was also performed to detect the neuronal apoptosis, endothelial continuity, and microglia accumulation. Western blot was performed to detect the protein expression of NEK7, NLRP3, ASC, caspase-1, IL-1β, BCL-2, and BAX.

### SAH Model

SAH model was performed as previously described (12); mice were anesthetized with pentobarbital sodium (40 mg/kg) by intraperitoneal (i.p.) injection, then exposed the bifurcation of the common carotid artery in supine position. The left external carotid artery (ECA) was isolated and distally cut into a 2-mm stump. A 5-0 sharpened monofilament nylon suture was inserted into the left internal carotid artery (ICA) through the ECA, then advanced 3 mm past the bifurcation of the left ICA, until resistance was felt. Sutures were withdrawn and ICA patency confirmed. In the sham group, sutures were not advanced past the internal carotid artery bifurcation, identical to other procedures.

### Intracerebroventricular Injection

The operation of intracerebroventricular injection was as previously described (13). A hole, lateral to the bregma 1.0 mm, was drilled on the skull without puncturing the dura. A needle of a 10-μL Hamilton syringe (Microliter 701; Hamilton Company, Reno, NV, USA) was then stereotactically and vertically inserted into the hole, 3.0 mm under the horizontal plane of the bregma, entering the left lateral ventricle. A total 2 μL volume of recombinant NEK7 protein solution (Sino Biological, Beijing, China, 4 μg dissolved in 2 μL sterile saline), nigericin solution (Sigma-Aldrich, Shanghai, China, 4 μg in 2 μL vehicle, vehicle: 2 μL mixture of 9:1 NS and DMSO), or MCC950 solution (Sigma-Aldrich, Shanghai, China, 2 μg in 2 μL vehicle, vehicle: 2 μL mixture of 9:1 NS and DMSO) was infused at a rate of 0.2 μL/min, 1 h after SAH induction, whereas 500 pmol/2 μL of NEK7 siRNA (Santa Cruz Biotechnology, Santa Cruz, CA, USA, 500 pmol in a 2-μL mixture of 1:1 RNase-free water and lipofectamine) or scrambled siRNA was infused at the identical rate 48 h before SAH induction. The syringe was slowly withdrawn at 10 min after infusion.

### Neurological Outcome Assessment

Neurological deficits were accessed as previously described, at 24 and 72 h after SAH induction. The modified Garcia scale and beam balance test were used (14). Two blinded observers were employed for grading mean of the neurological score.

### SAH Grade

The SAH severity grading score was implemented as previously described (15). The basal cistern was divided into six segments that could be scored from 0 to 3 according to the amount of subarachnoid blood clotting. The total score was calculated by adding the scores from six segments (0–18 points). Animals that received a score <8 were excluded from the study.

### Brain Water Content

The brain samples were quickly removed from the skull, separated into the left and right cerebral hemispheres, the cerebellum, and the brain stem. These four parts of the brain were weighed (wet weight), respectively, and the brain samples were then dried at 55°C for 48 h in an oven and weighed again (dry weight). The water content percentage formula was: ([wet weight - dry weight]/wet weight) × 100%.

### Evans Blue Extravasation

Evans blue extravasation was performed as previously described (14). Mice were anesthetized by pentobarbital sodium (40 mg/kg) i.p. injection 24 h post-SAH. Evans blue dye (2%, 5 mL/kg; Sigma–Aldrich, St. Louis, MO, USA) was injected into the left femoral vein over 2 min and circulated for 60 min. Mice were euthanized by an intracardial perfusion of phosphate-buffered saline (PBS). The brains were removed and quickly divided into the left and right cerebral hemispheres, weighed, homogenized in saline, and centrifuged at 15,000 g for 30 min. Subsequently, the resultant supernatant was added with equal volume of trichloroacetic acid, incubated overnight at 4°C and centrifuged at 15,000 g for 30 min. Next, the resultant supernatant was collected and spectrophotometrically quantified at 610 nm for Evans blue dye.

### Immunofluorescence Staining

Immunofluorescence staining was performed on fixed frozen brain sections as previously described (16, 17). Mice were deeply anesthetized and transcardially perfused with PBS and 4% PFA at 24 h post SAH. Brain samples were isolated and post-fixed in 4% PFA for 24 h, then soaked in 40% sucrose for 1 day. Coronal brain sections (10 μm) were obtained using a cryostat (Leica, Nussloch, Eisfeld, Germany) and permeabilized using 0.3% Triton X-100 in PBS for 30 min. Sections were blocked with 5% donkey serum for 1 and incubated at 4°C overnight with anti–Occludin (1: 100, Abcam, Cambridge, UK), anti-NEK7 (1: 200, Novus, Bio-Techne China, Shanghai, China), anti-IL-1β, anti–NeuN, anti–GFAP (1: 100), anti–Iba-1, anti–NG2 (1: 50, Abcam, Cambridge, UK) antibody and Lectin (1:200, Vector Laboratories, Burlingame, CA, USA), then incubated with corresponding secondary antibodies for 4 h at room temperature. Images were obtained atbasal cortex with a fluorescence microscope (Olympus, Melville, NY, USA) from slides. Quantification of immunofluorescence intensity was performed with the Measure tool in ImageJ software (National Institutes of Health, Bethesda, MD, USA) and the results of all the groups were normalized to the Sham group. Six image fields per group were analyzed. To evaluate the continuity of tight junctions of ECs, gaps of tight junctions were quantified as the length percentage of whole Occludin staining as per the previous method (18). The length of gaps and whole Occludin staining was measured by the Line tool in ImageJ software (National Institutes of Health, Bethesda, MD, USA) and the percentage was calculated as follows: Gap formation percentage = gap length/whole Occludin length. At least six vessels in each group were analyzed.

## Tunel

TUNEL staining was performed to test neuron apoptosis. Paraffin-embedded left/ ipsilateral hemisphere basal cortex sections (10 mm) were prepared. Apoptotic cells were stained using a Roche *in situ* cell death detection kit (Roche, Basel, Switzerland) according to the manufacturer's instructions. Fluorescent pictures were captured using a fluorescence microscope (Olympus, Melville, NY, USA). The number of TUNEL-positive cells were counted with ImageJ software. To calculate the apoptotic rates, six slides from one group were chosen and the number of TUNEL+ cells were counted in the range of images. The apoptotic rates were calculated by the formula: apoptotic rates = the number of TUNEL+ neurons in the field/ the number of all neurons in the field × 100%.

### Western Blot

Western blot was performed as previously described (19, 20). Briefly, a protein sample was extracted from the cortex of the left/ipsilateral hemisphere. Equal amounts of total protein (30 μg) were loaded in the lane of SDS-PAGE gels. After electrophoresis, the protein was transferred onto a PVDF membrane, which was then blocked with blocking buffer for 2 h at room temperature. Subsequently, the membrane was incubated at 4°C overnight with the following antibodies: anti-NEK7 (1:500; Novus, Bio-Techne China, Shanghai, China), anti-NLRP3, anti-ASC (1:500; Abcam, Cambridge, UK), anti-caspase-1, anti–IL-1β (1:500; Santa Cruz Biotechnology, Santa Cruz, CA, USA), anti-BCL-2, and anti-BAX (1:500; Thermo Fisher Scientific, Waltham, MA, USA). β-actin and α-tubulin were used as internal loading controls, anti–β-actin, and anti-α-tubulin primary antibodies (1:2000; Santa Cruz Biotechnology, Santa Cruz, CA, USA). PVDF membranes were incubated with the appropriate secondary antibodies for 2 h at room temperature. Blots were detected by chemiluminescent (ECL Plus; Amersham Bioscience, Arlington Heights, IL, USA). Data were analyzed by densitometry using Quantity One 4.6.2 (Bio-Rad Laboratories, Berkeley, CA, USA).

### Statistical Analysis

Data are shown as the mean ± SD. Chi-square tests and one-way ANOVA, followed by Tukey's multiple comparisons test were used to compare the different groups. SPSS 18 software (IBM, Chicago, IL, USA) was utilized to analyze the data, and *P* < 0.05 was considered as statistically significant.

## Results

### Expression of NEK7 and Cellular Distribution After SAH

**Figure 1:**
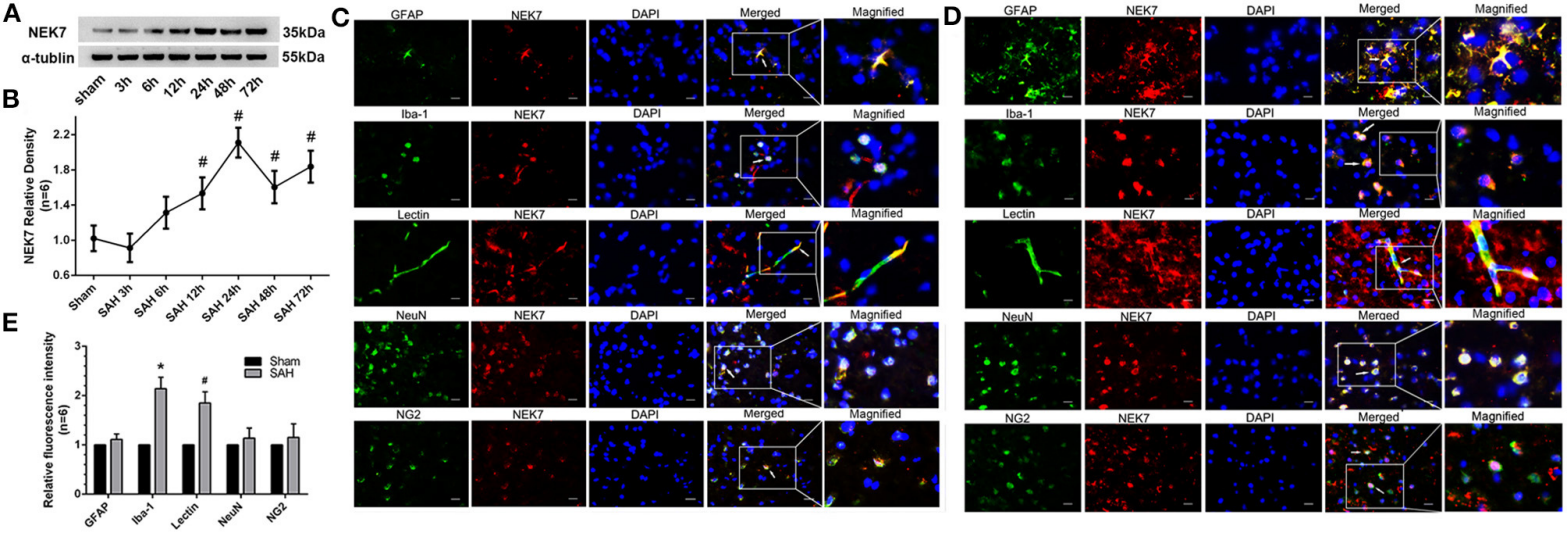
Time course of endogenous NEK7 and cell type location after subarachnoid hemorrhage. **(A,B)** Representative western blot bands and quantitative analysis of NEK7 from the ipsilateral cortex after SAH, *n* = 6. Relative densities of each protein have been normalized against the sham group. #: vs. sham *P* < 0.05. **(C,D)** Representative immunohistochemistry slices of NEK7 (red), GFAP, Iba-1, Lectin, NeuN, NG2, (green) in the sham and SAH group at 24 h after SAH, *n* = 1. Scale Bar = 10 μm. **(E)** Quantitative analysis of NEK7 fluorescent intensity in each type of cell. *: vs. sham (Iba-1) *P* < 0.05, #: vs. sham (Lectin) *P* < 0.05.

To investigate the expression of NEK7 after SAH induction, protein levels of NEK7 was detected by Western blot analysis. A significant increase of NEK7 were found in the ipsilateral/left hemisphere at 24 and 72 h after SAH (Figures 1A,B). Furthermore, immunohistochemical staining in the sham group showed that NEK7 expression was colocalized with the NeuN (neuron marker), GFAP (astrocyte marker), Lectin (endotheliocyte marker), Iba-1 (microglia marker), and nerve/glial antigen (NG) 2 (oligodendrocyte marker) in the left/ipsilateral cortex (Figure 1C). At 24 h post SAH, NEK7 was obviously elevated in endothelial cells and microglia (Figures 1D,E). None of the sham-operated mice died, and eight mice died within 72 h after SAH, due to severe hemorrhagic volume.

### Neurological Functions After Recombinant NEK7 Delivery and Interference of NEK7 Expression After SAH

**Figure 2:**
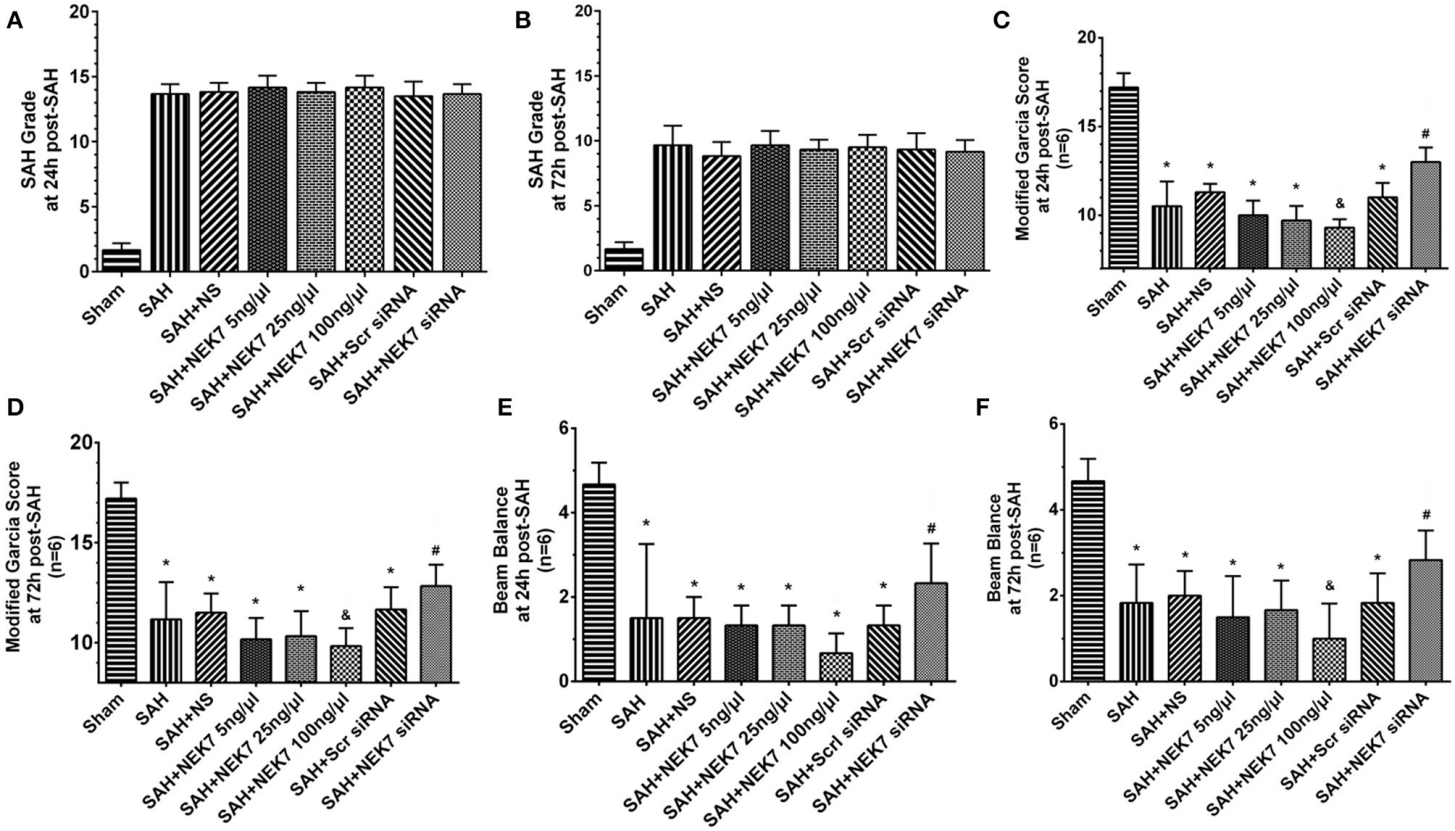
Effects of exogenous NEK7 and NEK7 small interfering RNA treatment on neurological outcomes after subarachnoid hemorrhage. **(A,B)** SAH grading score at 24 and 72 h after SAH **(C-F)** Modified Garcia and Beam balance test at 24 and 72 h after SAH, *n* = 6. *: vs. ham *P* < 0.05, &: vs. SAH+NS *P* < 0.05, and #: vs. SAH+Scr siRNA *P* < 0.05.

The NEK7 siRNA used in the present study could effectively inhibit the NEK7 expression after SAH. SAH grading score did not display significant differences among the groups at 24 and 72 h after SAH (Figures 2A,B). The SAH group showed significant neurological impairment in the modified Garcia test and beam balance, compared with the Sham group at 24 h after SAH induction (Figures 2C–F). Mice in the SAH+NEK7 100 ng/μL group had more severe neurological deficits at 24 h post SAH compared with the SAH+NS group, different to that in the SAH+NEK7 5 ng/μL and SAH+NEK7 25 ng group (Figures 2C–F). Furthermore, NEK7 siRNA treatment revealed greater improvements in neurological deficits compared with the SAH+Scr siRNA group at 24 h after SAH (Figures 2C–F). The Beam balance test exhibited similar trends (Figures 2C–F).

### Neuronal Apoptosis and BBB Permeability After Recombinant NEK7 Delivery and Interference of NEK7 Expression

**Figure 3:**
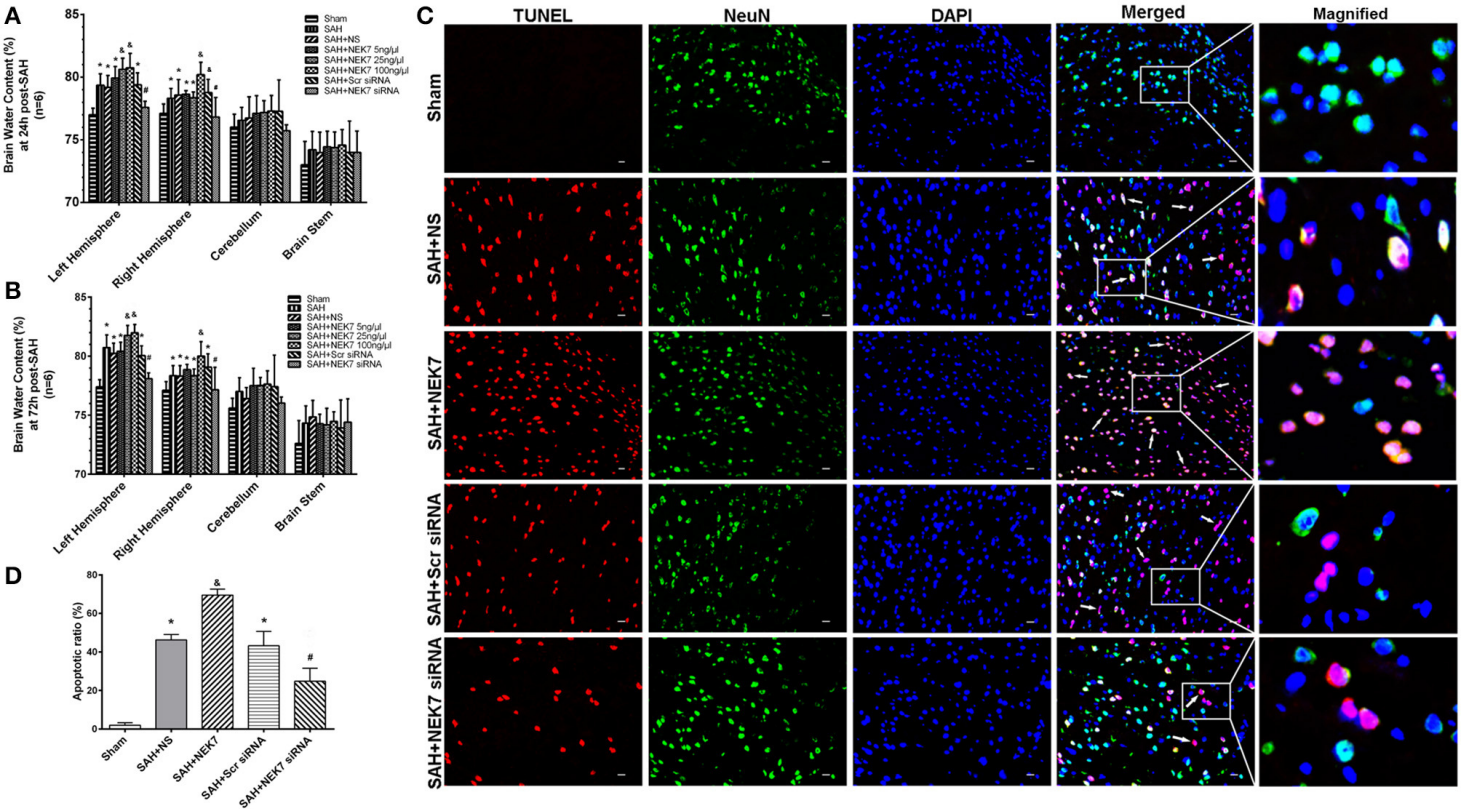
Effects of exogenous NEK7 and NEK7 small interfering RNA treatment on neuronal apoptosis after subarachnoid hemorrhage. **(A,B)** Brain water content assessment at 24 and 72 h after SAH, *n* = 6. **(C)** Representative immunohistochemistry images of TUNEL (red) and NeuN (green) at 24 h after SAH. Scale Bar = 20 μm. **(D)** Apoptotic ratios of each group, *n* = 6. *: vs. sham *P* < 0.05, &: vs. SAH+NS *P* < 0.05, and #: vs. SAH+Scr siRNA *P* < 0.05.

**Figure 4:**
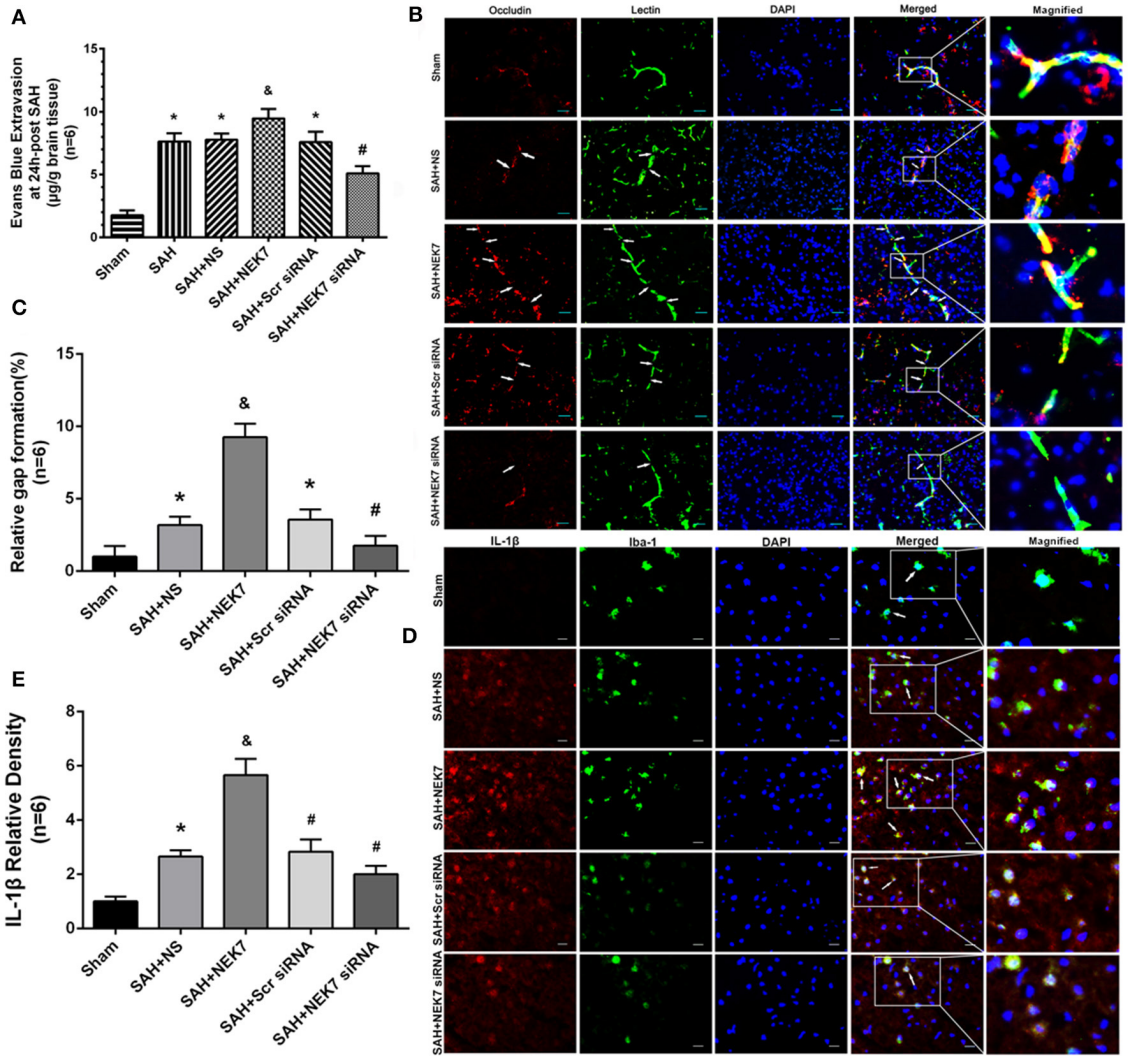
Effects of exogenous NEK7 and NEK7 small interfering RNA treatment on blood–brain barrier and microglia accumulation after subarachnoid hemorrhage. **(A)** Evans blue extravasation evaluation at 24 h after SAH, *n* = 6. **(B)** Representative immunohistochemistry images of Occludin (red) and Lectin (green) at 24 h after SAH. Arrow indicates the breakdown of continuous endothelia cell layer, *n* = 6. Scale Bar = 20 μm. **(C)** Quantitative analysis of endothelial gap in **(B)**. **(D)** Representative immunohistochemistry images of IL-1β (red) and Iba-1 (green) at 24 h after SAH. Arrow indicates the accumulation of microglia and overlap of IL-1β and Iba-1, *n* = 6. Scale Bar = 10 μm. **(E)** Quantitative analysis of IL-1β fluorescent in **(D)**. *: vs. sham *P* < 0.05, &: vs. SAH+NS *P* < 0.05, and #: vs. SAH+Scr siRNA *P* < 0.05.

Mice from the SAH, SAH+NS, and SAH+Scr siRNA groups showed increased brain water content in both hemispheres at 24 and 72 h after SAH compared with mice in the Sham group (Figures 3A,B). The NEK7 100 ng/μL treatment increased the brain water content compared with the SAH+NS group, while the NEK7 siRNA pretreatment significantly reduced the brain water content in both hemispheres at 24 and 72 h post SAH (Figures 3A,B). Therefore, a dosage of NEK7 100 ng/μL was chosen for further investigation. Apoptosis is a biologically reversible process which is characterized as energy-dependent programmed cell death (21). Neuronal cell apoptosis, a main process of EBI, plays a crucial role in pathophysiology after SAH (22). Mice in the SAH and SAH+NS group showed a significant increase of neuronal apoptosis in the left/ipsilateral cortex. In the SAH+NEK7 100 ng/μL group, mice exhibited a rising number of apoptosis neurons than that in the SAH+NS group, while NEK7 siRNA pretreatment reversed these phenomena (Figures 3C,D). Furthermore, the SAH group demonstrated more Evans blue extravasation than the Sham group in the left/ipsilateral hemisphere (Figure 4A). The SAH+NEK7 100 ng/μL mice exhibited increased Evans blue leakage compared with the SAH+NS mice, whereas the NEK7 siRNA pretreatment significantly lowered the Evans blue extravasation compared with the SAH+Scr siRNA group (Figure 4A). In the immunohistochemical staining images, continuous endothelial cells (Lectin) and Occludin in the sham group were shown. Th Lectin and Occludin continuous structures broke up at 24 h after SAH, and NEK7 treatment significantly aggravated those damages, however, NEK7 siRNA infusion significantly reversed those disruptions at 24 h after SAH (Figures 4B,C). There was a significant accumulation of microglia in the SAH+NS group compared to that in the sham group (Figure 4D). IL-1β expression in microglia was elevated in the SAH+NS group after SAH and was aggravated by NEK7 infusion, while NEK7 siRNA administration reversed those effects (Figure 4E).

### NLRP3 Inflammasome Protein and Apoptosis Related Protein Alteration After Recombinant NEK7 Delivery and Specific Inhibition of NEK7 Expression

**Figure 5:**
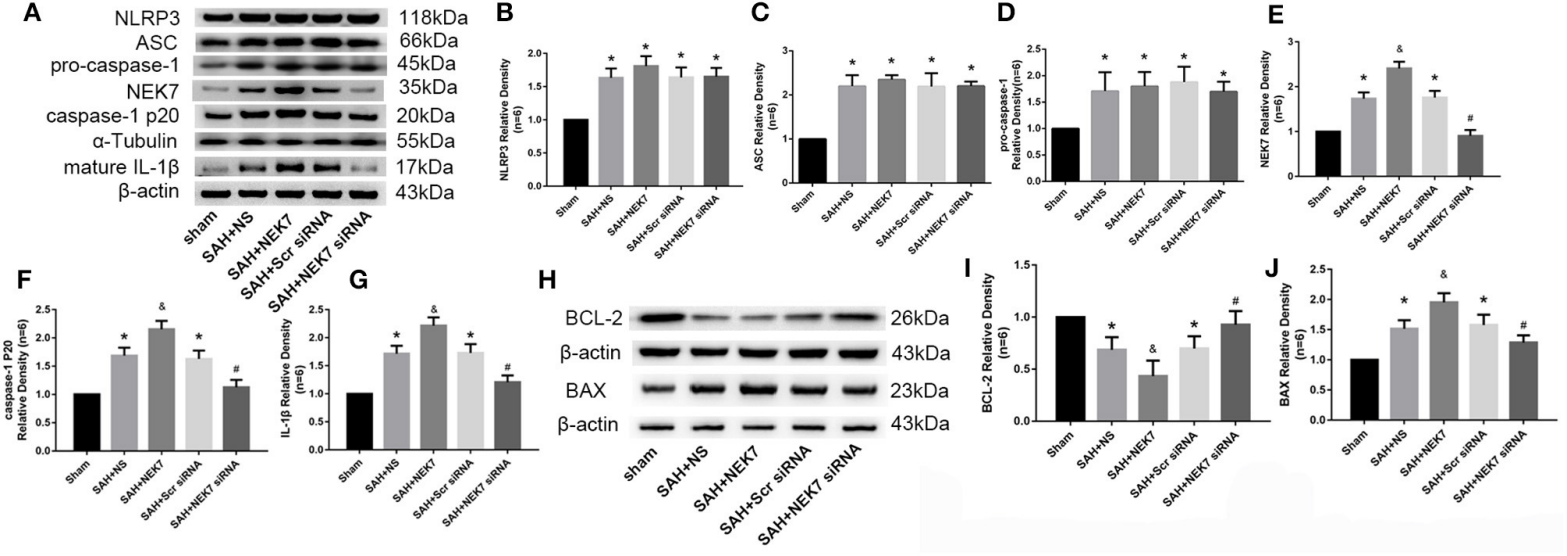
Effects of exogenous NEK7 and NEK7 small interfering RNA pretreatment on NLRP3 activation and apoptosis related protein expression. **(A)** Representative western blot bands of NLRP3, NEK7, caspase-1, ASC, and IL-1β from the ipsilateral cortex after SAH. **(B–G)** Quantitative analysis of NLRP3, ASC, pro-caspase-1, caspase-1 p20, NEK7, and IL-1β, *n* = 6. **(H)** Representative western blot bands of BCL-2 and BAX. **(I,J)** Quantitative analysis of BCL-2 and BAX. Relative densities of each protein have been normalized to the sham group. *n* = 6. *: vs. sham *P* < 0.05, &: vs. SAH+NS *P* < 0.05, and #: vs. SAH+ scrambled siRNA *P* < 0.05.

Expression of NEK7, NLRP3, ASC, caspase-1, and IL-1β was measured *via* western blot at 24 h after SAH. NLRP3 levels in SAH, SAH+NS, SAH+NEK7 100 ng/μL, SAH+Scr siRNA, and SAH+ NEK7 siRNA elevated significantly at 24 h post SAH (Figures 5A,B). NEK7 100 ng/μL infusion after SAH increased ASC expression and caspase-1 and IL-1β maturation, however, NEK7 siRNA pretreatment reduced mature caspase-1 and IL-1β expression (Figures 5A–G). BCL-2 (B-cell lymphoma 2), is a member of the BCL-2 family of regulator proteins that inhibit apoptosis. It plays an important role in promoting cellular survival and inhibiting the actions of pro-apoptotic proteins (21). BAX (Bcl-2-associated X protein) is a pro-apoptotic protein in the BCL-2 family, which acts on the mitochondrial membrane to promote permeabilization, which is a vital signal in the apoptosis cascade (23). Mice from the SAH and SAH+NS groups showed significantly increased levels of BAX and decreased BCL-2 (Figures 5H–J). In the SAH+NEK7 group, expression of BAX rose and BCL-2 reduced, compared to that in the SAH+NS group. While in the SAH+NEK7 siRNA group, expression of BAX was lower, and BCL-2 was more than that in the SAH+Scr siRNA group (Figures 5H–J).

### Neurological Functions and Neuronal Apoptosis After Nigericin Delivery, Specific Inhibition of NEK7 Expression, and MCC950 Administration

**Figure 6:**
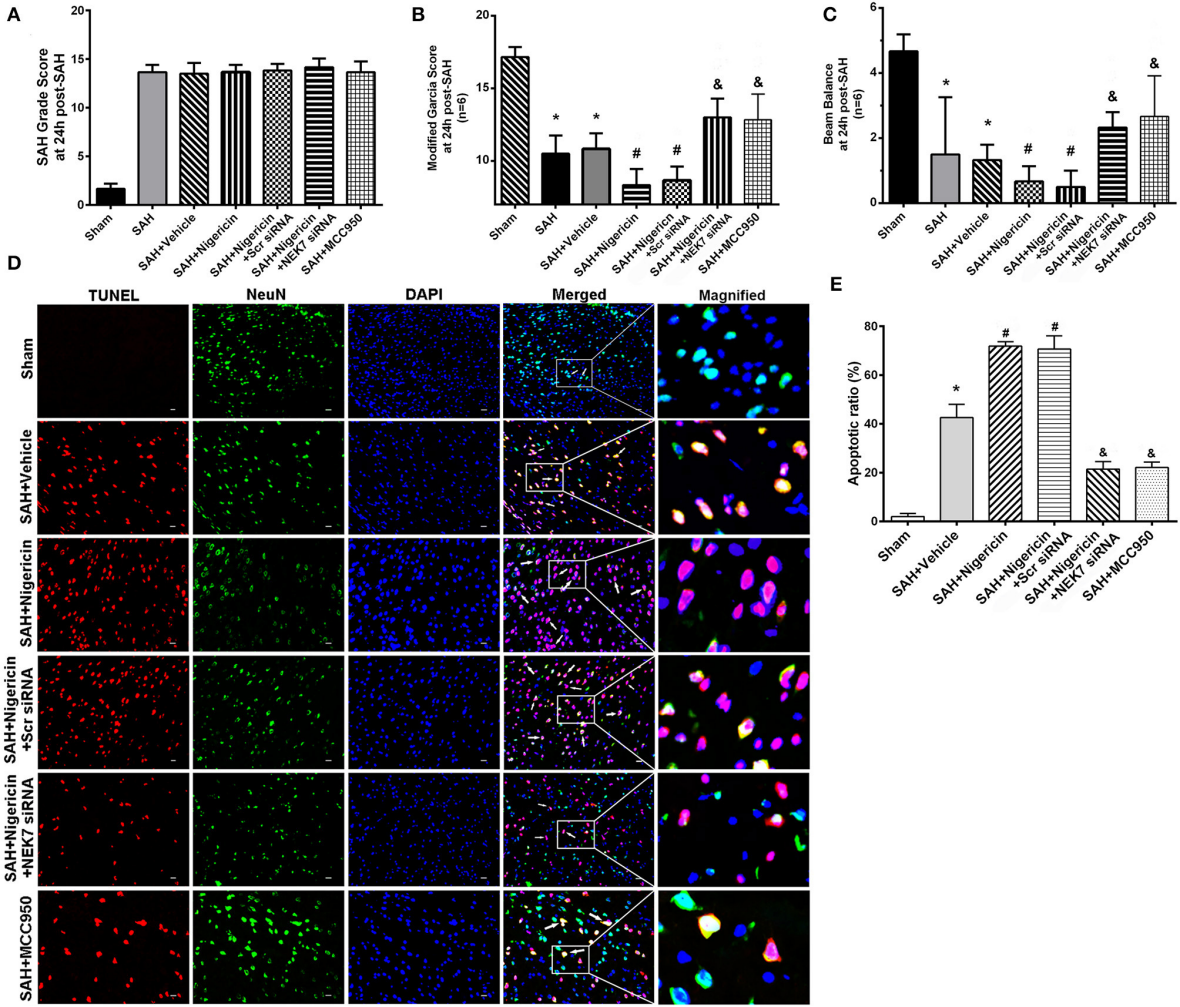
Effects of nigericin, NEK7 small interfering RNA and MCC950 treatment on neurological outcome. **(A)** SAH grading score at 24 and 72 h after SAH **(B,C)** Modified Garcia test and Beam balance test at 24 h after SAH. *n* = 6 for each group. **(D)** Representative immunohistochemistry images of TUNEL (red) and NeuN (green) at 24 h after SAH. Scale Bar=20 μm. **(E)** Apoptotic ratios of each group, *n* = 6. *: vs. sham *P* < 0.05, &: vs. SAH+Vehicle *P* < 0.05, and #: vs. SAH+nigericin+Scr siRNA *P* < 0.05.

To study whether NLRP3 activation is dependent on NEK7 after SAH, we used nigericin, an activator of NEK7-dependent NLRP3 signal (10), or MCC950, an inhibitor of the NEK7/NLRP3 signal (24), to regulate inflammation post SAH. The SAH grading score did not show significant differences among the groups at 24 h post SAH (Figure 6A). The SAH and SAH+Vehcile group showed significant neurological impairment in the modified Garcia score and beam balance test compared with the Sham group (Figures 6B,C). Mice in the SAH+negricin group had more severe neurological deficits at 24 h post SAH compared with the SAH+Vehicle group (Figures 6A–C). NEK7 siRNA treatment or MCC950 ameliorated neurological deficits, however, were comparable to that of the SAH+Scr siRNA group or SAH+Vehicle group, respectively (Figures 6A–C). Compared with the Sham group, the SAH+Vehicle group displayed obvious increasing apoptosis neuron numbers at the left/ipsilateral cortex at 24 h post SAH (Figures 6D,E). Nigericin administration increased neuronal apoptosis after SAH, while NEK7 knockdown or MCC950 injection reduced neuronal apoptosis (Figures 6D,E).

### BBB Permeability After Nigericin or MCC950 Delivery and Specific Inhibition of NEK7 Expression

**Figure 7:**
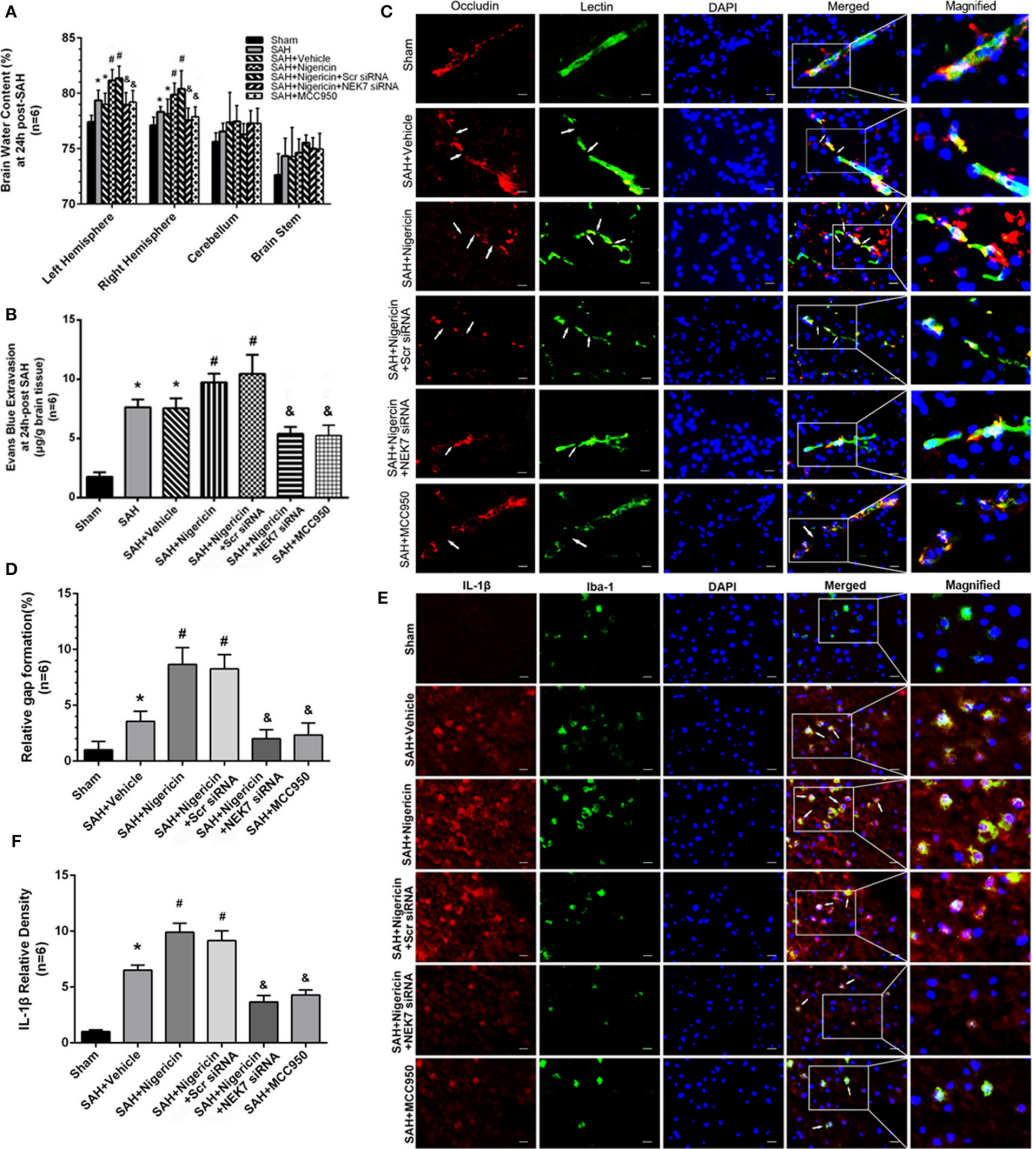
Effects of nigericin, NEK7 small interfering RNA and MCC950 treatment on blood–brain barrier and microglia accumulation after subarachnoid hemorrhage. **(A)** Brain water content assessment at 24 h after SAH, *n* = 6. **(B)** Evans blue extravasation evaluation at 24 h after SAH, *n* = 6. **(C)** Representative immunohistochemistry images of Occludin (red) and Lectin (green) at 24 h after SAH. Arrow indicates the breakdown of continuous endothelia cell layer, *n* = 6. **(D)** Quantitative analysis of endothelial gap in **(C)**. **(E)** Representative immunohistochemistry images of IL-1β (red) and Iba-1 (green) at 24 h after SAH. Arrow indicates the accumulation of microglia and overlap of IL-1β, *n* = 6. Scale Bar = 10 μm. **(F)** Quantitative analysis of IL-1β intensity in **(E)**. *: vs. sham *P* < 0.05, &: vs. SAH+Vehicle *P* < 0.05, and #: vs. SAH+nigericin+Scr siRNA *P* < 0.05.

Mice in the SAH and SAH+Vehicle groups showed increased brain water content in left hemispheres 24 h after SAH, compared with the Sham group (Figure 7A). The nigericin treatment increased the brain water content compared with the SAH+ Vehicle group, however the NEK7 siRNA pretreatment or MCC950 injection significantly reduced the brain water content (Figure 7A). Furthermore, the SAH group showed more Evans blue extravasation than the Sham group (Figure 7B). The SAH+Nigericin group exhibited increased Evans blue leakage compared with the SAH+Vehicle group, whereas NEK7 siRNA pretreatment or MCC950 delivery significantly lowered the Evans blue extravasation compared with the SAH+Nigericin+Scr siRNA group (Figure 7B). Lectin and Occludin continuous structures broke up at 24 h after SAH, and nigericin treatment significantly aggravated those damages, while NEK7 siRNA injection significantly reversed those disruptions (Figure 7C). Microglia accumulation was exacerbated and expression of IL-1β in microglia was elevated *via* nigericin administration and was alleviated after NEK7 siRNA pretreatment or MCC950 injection (Figures 7D,E).

### NEK7 Coordinated NLRP3 Signal Activation After SAH

**Figure 8:**
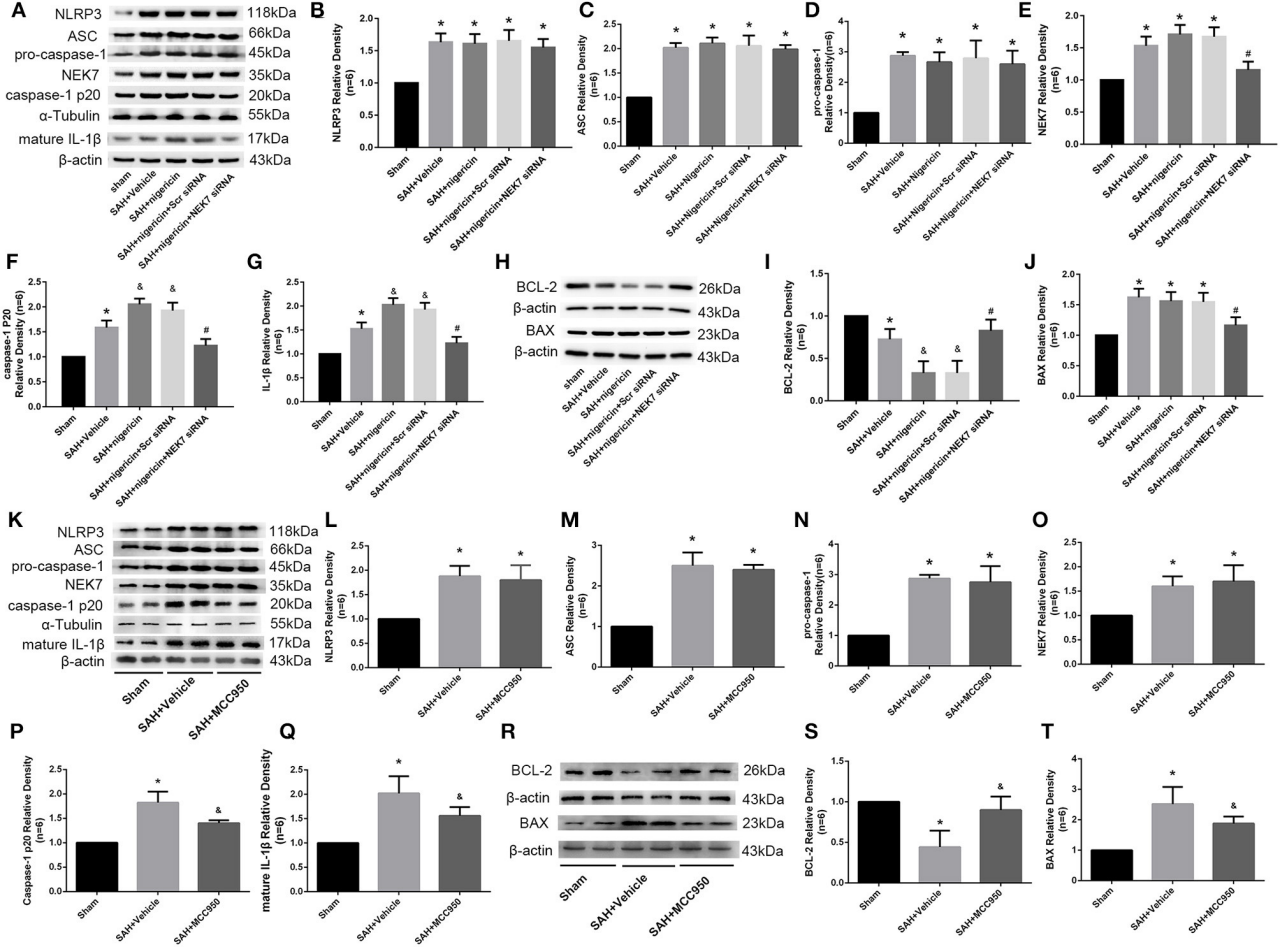
Effects of nigericin, NEK7 small interfering RNA pretreatment, and MCC950 treatment on NLRP3 activation and apoptosis related protein expression. **(A,K)** Representative western blot bands of NLRP3, NEK7, ASC, caspase-1, and IL-1β from the ipsilateral cortex after SAH. **(B–G, L–Q)** Quantitative analysis of NLRP3, ASC, pro-caspase-1, caspase-1 p20, NEK7 and IL-1β, *n* = 6. **(H,R)** Representative western blot bands of BCL-2 and BAX. **(I,J,S,T)** Quantitative analysis of BCL-2 and BAX. Relative densities of each protein have been normalized to the sham group, *n* = 6. *: vs. sham *P* < 0.05, &: vs. SAH+Vehicle *P* < 0.05, and #: vs. SAH+nigericin+Scr siRNA *P* < 0.05.

Expression of NEK7, NLRP3, ASC, pro-caspase-1, caspase-1 and IL-1β was measured *via* western blot at 24 h after SAH. NLRP3 was activated by various stimuli, and we wondered whether NEK7 coordinates NLRP3 activation after SAH. The expression of NEK7, NLRP3, caspase-1, and IL-1β was significantly increased at 24 h after SAH and NEK7 expression peaked at 24 h (Figures 5A–G). NEK7 recombinant protein delivery increased ASC oligomerization, caspase-1, and IL-1β maturation without increasing NLRP3 expression, and this was reversed by NEK7 siRNA infusion or MCC950 injection, which indicated that increasing NEK7 and NLRP3 binding activated the downstream signal (Figures 5A–G). BCL-2 protein levels in the SAH group were decreased at 24 h after SAH compared to the Sham group, while BAX increased and BCL-2 decreased in the SAH+Vehicle group compared to the sham group (Figures 5H–J). NEK7 recombinant protein infusion lowered BCl-2 and elevated BAX, while NEK7 siRNA relieved those effects (Figures 5H–J). Nigericin injection increased the expression of caspase-1, IL-1β at 24 h after SAH, which demonstrated that nigericin increased caspase-1 and IL-1β maturation but did not affect NLRP3 and NEK7 expression (Figures 8A–G,K–Q). Caspase-1 and IL-1β maturation were decreased at 24 h after SAH in the SAH+nigericin+Scr siRNA group compared to that in the SAH+nigericin+NEK7 siRNA group (Figures 8A–G,K–Q). Nigericin treatment could effectively inhibit their BCL-2 expression and enhance BAX expression (Figures 8H–J), however NEK7 siRNA treatment (Figures 8H–J) or MCC950 administration significantly reversed those trends (Figures 8R–T).

## Discussion

The present study demonstrated that NEK7 increased after SAH, peaked at 24 h, and was mainly expressed in microglia and endothelial cells. Exogenous NEK7 protein worsened neurological deficits and brain edema, while NEK7 specific inhibition preserved neurological function, neuronal apoptosis, and brain edema. Administration of Nigericin, a NEK7-dependent NLRP3 activator, aggravated neurological deficits, brain edema, microglial accumulation, and BBB disruption, while NEK7 siRNA infusion reversed these phenomena. By further studying the mechanism it was indicated that NEK7 activated the NLRP3 downstream inflammatory pathway, including caspase-1 and IL-1β maturation in endothelial cells which open, leading to increasing BBB extravasation. The microglia were then activated, which subsequently induced neuronal apoptosis by reducing BCL-2 and increasing BAX. NEK7 was recently reported to be critical for the activation of NLRP3 inflammasome and its downstream inflammatory pathway, to induce caspase-1 and IL-1β maturation (4, 10, 25). NLRP3 inflammasome, which is important for the activation of caspases-1, maturation, and the secretion of proinflammatory cytokines such as IL-1β and IL-18, is a complex with multi-protein components containing three parts: an amino-terminal pyrin domain, a central nucleotide-binding domain, and a C-terminal leucine rich repeat (26). The ASC, and pro-caspase-1 are closely linked with the formation of NLRP3 inflammasome (27). Under the pathological state, NLRP3 is activated, then inflammasome assembly takes place and leads to the IL-1β shearing from pro-IL-1β, then secretion and maturation, which finally causes cell injury and death (7). NLRP3 inflammasome is involved in the initiation and development of several pathophysiological processes of CNS injury including stroke (28), neurodegenerative disease (7), and multiple sclerosis (29).

NEKs (NIMA-related kinase) are a group of evolutionarily conserved protein kinases sharing high amino acid sequence identity with NIMA which controls initiation of cell mitosis during mitotic cell division (30). A decrease in the centrosomal c-tubulin levels and reduction of the microtubule re-growth activity in the NEK7-suppressed cells was observed (31). The NEK7 amino acid sequence contains all of the amino acids conserved in serine/threonine kinases and have no C-terminal extensions (32), is expressed widely in a mouse brain including the Olfactory bulb, cortex, Thalamus, Midbrain, and the Hindbrain (33), is considered to be a new inflammasome component, and is also a switch between mitosis and NLRP3 activation (11). A recent study showed that NEK7-mutant mice had a defective IL-1β production response to nigericin (11). NEK7 bound to the leucine-rich repeat domain of NLRP3 in a kinase-independent manner (25) and acted as a coordinator in NLRP3 inflammasome activation. NLRP3 inflammasome assembly and formation is required for NEK7, which results in the secretion and maturation of IL-1β and IL-18 (11). MCC950 is an orally bioavailable sulfonylurea derived small molecule which selectively interacts with NLRP3 with no effect on NLRC4 and NLRP1 (34), and a number of studies showed its ability to block NLRP3 activation through the NEK7/NLRP3 pathway under pathological conditions (24), however, the precise mechanism is unknown. Some studies have demonstrated that MCC950 could reduce lung ischemia-reperfusion injury and high level of glucose induced retina damage by blocking NEK7-NLRP3 interaction (35, 36). Here we used MCC950 to block NEK7-NLRP3 pathway activation, and found that MCC950 showed similar effects as described in previous publications (37, 38). However, how MCC950 influences the NEK7-NLRP3 pathway still requires further study. Several pathways are pivotal for the NLRP3 inflammasome activation, such as potassium efflux, ROS (reactive oxygen species) signaling, and lysosomal destabilization. 50 mM KCl prevented potassium efflux to inhibit the interaction between NLRP3 and NEK7 induced by ATP, nigericin, or gramicidin in bone-marrow-derived macrophages from C57BL/6J mice carrying mutant NEK7 (25). While LPS-primed NEK7^+/+^or NEK7^Cu/Cu^ macrophages, producing similar amounts of mitochondrial ROS after treatment with nigericin (11). A recent pilot study found that hypokalemia is common in SAH patients (39). Cortical spreading depolarization, a phenomenon which is characterized as serial neuronal exciting *via* K^+^ channel open after SAH, was considered as a factor of poor prognosis (40). These studies suggested that K^+^ efflux may exist after SAH and could be an activator of NEK7/NLRP3. Recently, NEK7 has been identified as a necessary component of the NLRP3 activation driven by ROS but is independent of K+ efflux (41). The upstream of NEK7/NLRP3 activation after SAH currently remains elusive and requires further investigation.

Microglia are a cluster of specialized macrophages residing in the CNS system and the main source of NLRP3 (42). Sufficient evidence suggests that they play a critical role in neuroinflammation. Activated microglia produce and secrete pro-inflammatory cytokines, including IL-1β, nitric oxide, and tumor necrosis factor-α, which induce cell death and secondary central nervous system injury. Cultured microglia have recently been shown to produce IL-1β (11), acting as a key mediator of neuronal apoptosis after SAH (43). In addition, Microglia were found to induce neuronal apoptosis *via* reducing BCL-2 and increasing BAX (44). In this study, we showed that NEK7 was expressed in microglia and was elevated after SAH, which in turn elicited neuronal apoptosis by activating NLRP3 and inducing IL-1β releasing. Neuronal apoptosis was alleviated by NEK7 siRNA pretreatment *via* reducing IL-1β and BAX and increasing BCL-2. Microglial accumulation was also found after SAH, providing evidence that targeting NEK7 may reduce neurological deficits by attenuated neuronal apoptosis triggered by microglia activation. Our results displayed that microglial accumulation in the ipsilateral/left cortex was enhanced by exogenous NEK7 administration and nigericin infusion, while it was reversed by NEK7 siRNA pretreatment and MCC950 injection. NEK7 was reported to influence microtubule dynamics, which is also a main factor for cell movement, morphology maintaining, and synaptic outputs of neurons (31, 45, 46). Microtubule activity were also found to be associated with microglia migration (47), however, there are very few studies that mention the exact mechanism of microglia movement after SAH. Based on these results, NEK7 may play a potential role in microglia migration *via* regulating microtubule. Furthermore, Acetylated α-tubulin was recently found to mediate the transport of mitochondria, apposition of ASC to NLRP3 on endoplasmic reticulum, and then activated NLRP3 (48). This indicates that NEK7 might activate NLRP3 *via* activating microtubule acetylation after stroke, which requires further investigation.

The blood-brain barrier can prevent harmful substances from coming into contact with the brain and allows nutrient substances to enter the brain (49). After SAH, the blood-brain barrier opens and leads to blood metabolites coming into direct contact with neurons and surrounding cells, which also leads to vasogenic brain edema (2). Endothelial cells are a main component of the BBB. Endothelial cells maintain the basic function of the BBB though transcellular transport and a tight junction structure. Previous studies indicated that NLRP3 activation induce the BBB open, while NLRP3 deletion preserved BBB permeability by reducing MMP9 and MMP2, which could subsequently degrade the tight junction protein including zonula occludens-1 and TJP2 (50). Expression of NLRP3 rise in endothelial cells after stroke, suggesting that endothelial cells may be a major source of NLRP3 (36, 50). Recent studies reveal that knockdown NEK7 expression could reduce high glucose induced retinal endothelial cell dysfunction (36) and maintain retinal endothelial cell permeability, which suggests that IL-1β itself could increase BBB permeability. In this investigation we demonstrated that brain edema worsened, and Evans blue extravasation increased after SAH, and protein levels of IL-1β rose in endothelial cells. At the same time, knockdown NEK7 ameliorated downstream NLRP3 activation and IL-1β maturation, eventually protecting BBB.

The limitations of this investigation were that data only focused on the NEK7 and NLRP3 inflammasome, while tight junction protein degradation was not studied in detail. After SAH, NF-κB undergoes nuclear translocation, and subsequently, elevated MMP9 transcription and induced basement membrane and tight junction protein degradation, which have all been well-illustrated in other studies. In addition, NEK7 may be located in the extracellular region and could affect surrounding cells. Consistent with previous studies, we found that endothelial cells and microglia highly expressed NEK7, however, whether NEK7-NLRP3 pathway activation in other cells and whether other types of cells secrete NEK7 and have effects on endothelial cells and microglia after SAH, are unknown. Other unknown mechanisms of NEK7 or NLRP3 activation cannot be excluded, therefore, in further studies, more data about the expression of NEK7 in other cell types and the elucidation of mechanisms of other mechanisms are needed.

## Conclusions

**Figure 9:**
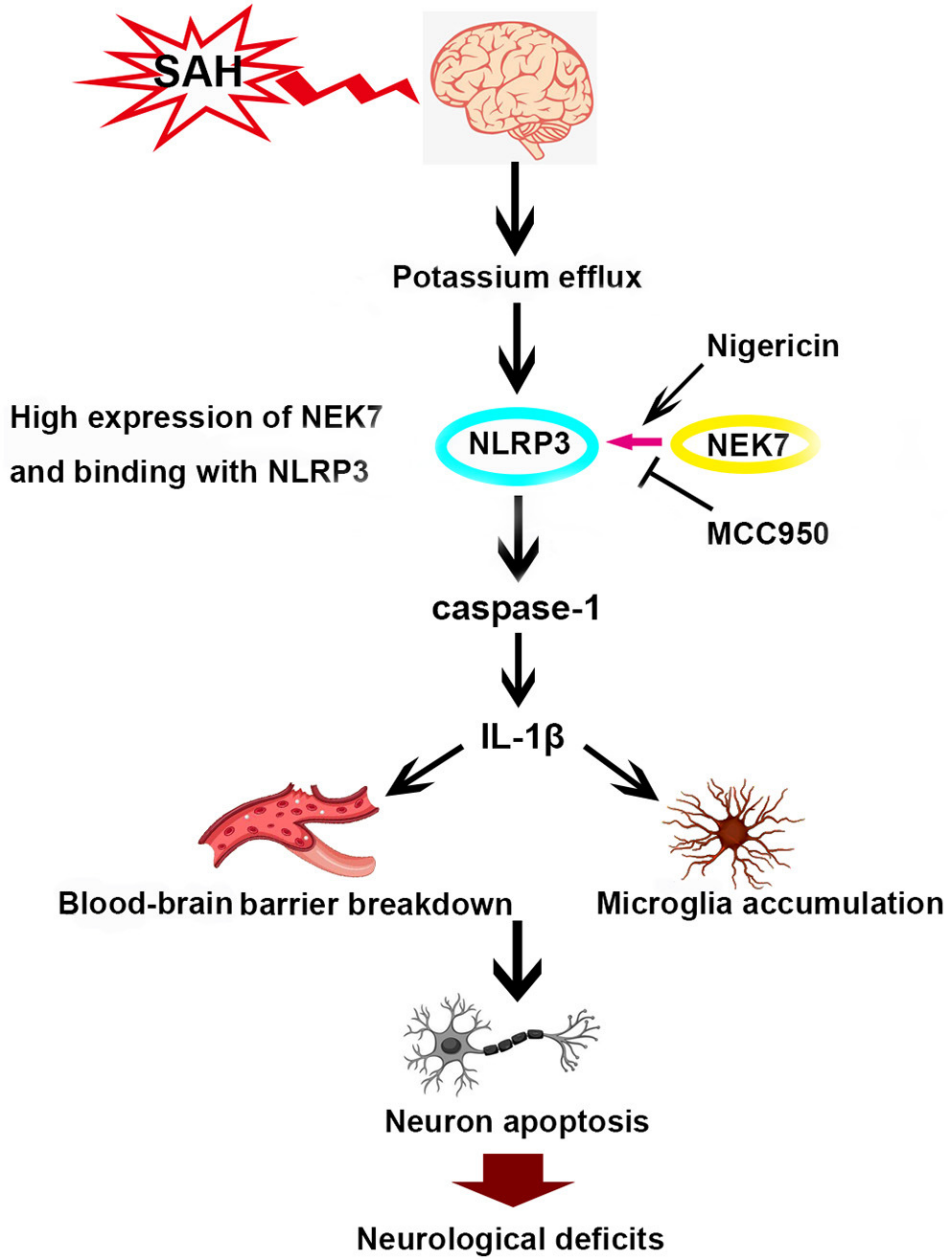
Scheme of NEK7/NLRP3 interaction and downstream signal activation after SAH.

This study demonstrated for the first time that NEK7 was elevated in the microglia and endothelial cells of the brain after SAH, and exogenous recombinant NEK7 induced neuronal apoptosis and blood–brain barrier disruption which subsequently worsens the neurological outcome. This process was potentially mediated by NEK7-NLRP3 activation, which may promote casepase-1 and IL-1β maturation and secretion, and apoptosis related protein alteration in the brain (Figure 9). By targeting NEK7, our data and further translational studies may provide benefits for the management of SAH patients.

## Data Availability Statement

All datasets generated for this study are included in the article/supplementary material.

## Ethics Statement

The animal study was reviewed and approved by Ethic Committee of General hospital of Northern Theater Command.

## Author Contributions

YD, GLia, and PP designed the experiment. PP, GLi, DL, ZZ, and GH finished the experiments protocols. GLi and DL carried out data analysis. PP, YD, and GLia prepared and revised the manuscript and gave the final approval of manuscript to be published. All authors contributed to the article and approved the submitted version.

## Conflict of Interest

The authors declare that the research was conducted in the absence of any commercial or financial relationships that could be construed as a potential conflict of interest.

## Abbreviations

ANOVA: analysis of variance
ASC: apoptosis-associated speck-like protein
BBB: blood–brain barrier
BAX: BCL-2-associated X protein
BCL-2: B-cell lymphoma 2
EBI: early brain injury
IL-1β: Interleukin-1 beta
LRR: leucine rich repeat
NBD: nucleotide-binding domain
NEK7: Never in mitosis gene A-related expressed kinase 7
NLRP3, NLR family: pyrin domain containing 3
PBS: phosphate-buffered saline
PYD: pyrin domain
SAH: subarachnoid hemorrhage
TJs: tight junctions
ZO-1: zonula occludens-1.
